# How anthropogenic shifts in plant community composition alter soil food webs

**DOI:** 10.12688/f1000research.13008.1

**Published:** 2018-01-02

**Authors:** Paul Kardol, Jonathan R. De Long

**Affiliations:** 1 Department of Forest Ecology and Management, Swedish University of Agricultural Sciences, Uppsala, Sweden; 2 Department of Terrestrial Ecology, Netherlands Institute of Ecology, Wageningen, Netherlands

**Keywords:** anthropogenic, soil, biodiversity

## Abstract

There are great concerns about the impacts of soil biodiversity loss on ecosystem functions and services such as nutrient cycling, food production, and carbon storage. A diverse community of soil organisms that together comprise a complex food web mediates such ecosystem functions and services. Recent advances have shed light on the key drivers of soil food web structure, but a conceptual integration is lacking. Here, we explore how human-induced changes in plant community composition influence soil food webs. We present a framework describing the mechanistic underpinnings of how shifts in plant litter and root traits and microclimatic variables impact on the diversity, structure, and function of the soil food web. We then illustrate our framework by discussing how shifts in plant communities resulting from land-use change, climatic change, and species invasions affect soil food web structure and functioning. We argue that unravelling the mechanistic links between plant community trait composition and soil food webs is essential to understanding the cascading effects of anthropogenic shifts in plant communities on ecosystem functions and services.

## Introduction

The soil food web consists of a large diversity of organisms differing in size and function. This includes root-associated biota such as pathogens or mutualists, saprotrophs involved in breaking down dead organic matter, and a variety of invertebrate consumers and predators at higher trophic levels ^1– 3^. As increasingly evidenced by empirical studies, soil food webs play a key role in the functioning of terrestrial ecosystems ^4– 7^. Soil food webs affect carbon (C) cycling (with consequences for C storage and hence mitigation of elevated atmospheric carbon dioxide concentrations) and nutrient cycling. On the one hand, soil food webs play an important role in controlling the supply of nitrogen (N) to plants by mineralizing organic N. However, N mineralized through the soil food web does not necessarily result in nutrients freely available for plants ^8^. Soil food webs can promote retention of N in the soil system either directly through sequestration in their living or dead biomass or indirectly through changes to soil chemistry or structure, thereby preventing it from getting lost through leaching and denitrification. It has been shown how shifts in the composition, network structure, and connectivity of soil food webs can alter the rates of these important ecosystem processes ^4, 7– 10^. The soil food web further plays an important role in disease suppression and plant protection against root pathogens ^11, 12^. Finally, the soil food web is critical to ecosystem resistance and resilience against environmental disturbances and climate change. For example, studies have shown that fungal-based soil food webs associated with extensively managed grasslands (that is, managed with minimal capital, labor, and artificial inputs) were more resistant to experimental drought than bacterial-based food webs associated with intensively managed crop production ^13^. Collectively, these recent advances indicate that changes in soil food web composition and connectivity have important consequences for ecosystem functioning ^14^.

**Figure 1.**
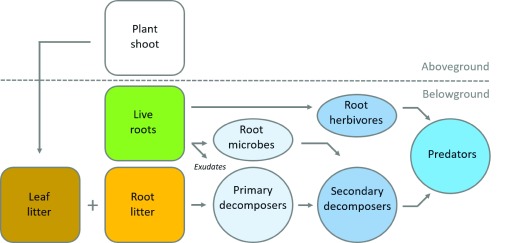
Simplified conceptual illustration of pathways of plant resource input to the soil food web. Most of the carbon fixed in plant tissue enters the soil in the form of dead roots and leaves or via the exudation of organic compounds from the roots. These inputs form a food source for detritus feeders and primary decomposers (bacteria and fungi). Living plant roots provide a food source for root-associated microbes (pathogens, nitrogen-fixing bacteria and mycorrhizal fungi) and root herbivores (root-feeding insect larvae and plant-feeding nematodes). Primary decomposers are fed upon by secondary decomposers (such as protozoa, microbial-feeding nematodes, collembola, and mites). Finally, secondary consumers as well as root herbivores are preyed upon by predators (such as predatory mites and centipedes).

Although most soil food webs are highly complex, comprising a plethora of feeding relationships, including high levels of omnivory ^15^, soil food webs are often simplistically described in terms of distinct trophic levels. Trophic levels are composed of organisms that occupy the same level in a food chain. In the soil food web, this would be primary consumers (for example, bacteria and fungi), secondary consumers (for example, microbial-feeding nematodes), and higher-level consumers or predators (for example, centipedes and predatory mites) ( Figure 1). Each of these trophic levels can be composed of a large taxonomic and functional diversity of organisms ^5^. A key question here is what are the main drivers of the structure and functioning of soil food webs. Macroclimate and biogeographical influences may constrain the pool of soil species from which local soil food webs assemble ^16^, but it is likely that resource availability plays a larger role in shaping soil food web structure, particularly at local scales. In most natural terrestrial ecosystems, about 80–90% of the C fixed in plant tissue ultimately enters the soil in the form of dead leaves and roots or via root exudation (that is, the release of organic compounds from the roots into the soil). These inputs form the basal resource pool for the soil food web ^17, 18^ ( Figure 1). Although studies have focused primarily on the input of aboveground plant litter (that is, leaves), more recently it has been shown that the input of root litter might be equally important and, interestingly, could have differential effects on the soil food web ^19, 20^. Furthermore, living plant roots provide the food source for root-feeding insects and nematodes and other root-associated biota such as mycorrhizal fungi ^19, 21^.

## Channels through which plant communities affect soil food webs

**Figure 2.**
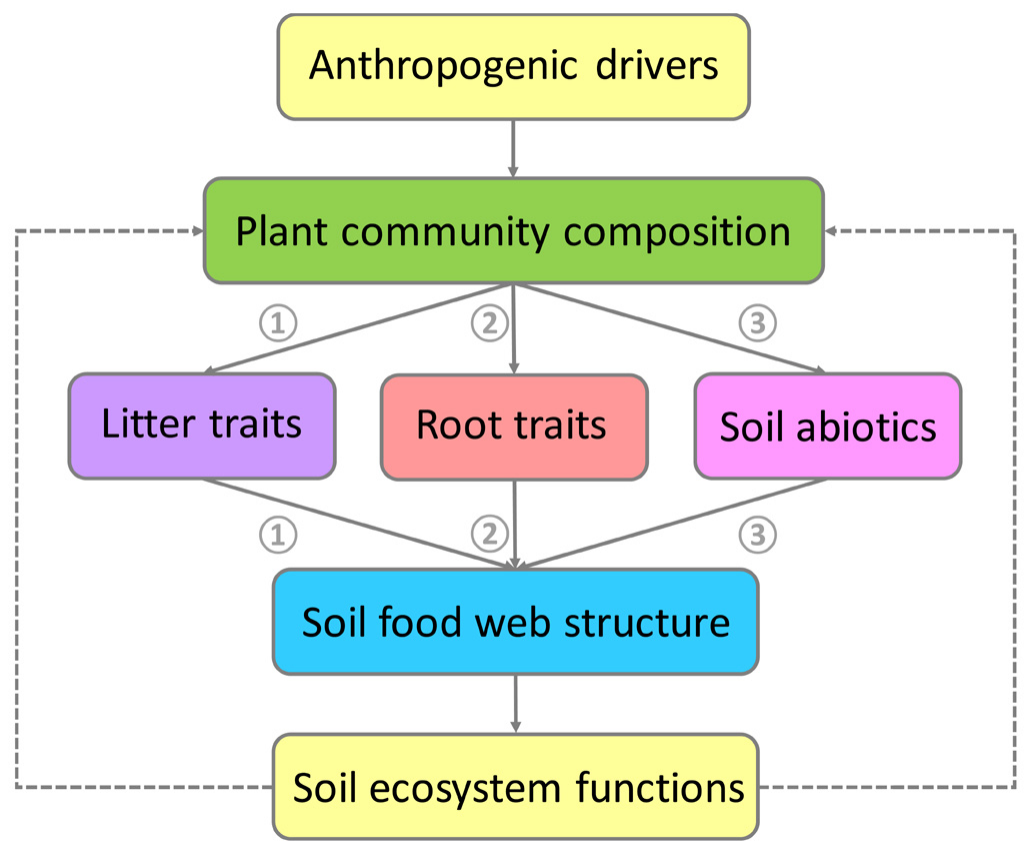
Conceptual diagram of pathways of how shifts in plant community composition affect soil food webs. Natural and anthropogenic shifts in plant community composition can impact on soil food webs by changes in the quantity and chemical composition of litter (that is, dead plant tissue, shoots, and roots) (pathway 1); by changes in root morphology, tissue chemistry, and composition of exudates (pathway 2); or by changes in soil abiotic conditions, such as availability of resources (for example, nutrients and water) and microclimate (for example, temperature) (pathway 3). Changes in soil food web structure as mediated by shifts in plant community composition have important consequences for soil ecosystem functions, such as carbon and nutrient cycling and disease suppression. In turn, changes in soil ecosystem functioning can feed back to plant community composition (dotted line); feedback effects are not a focus of this article.

**Figure 3.**
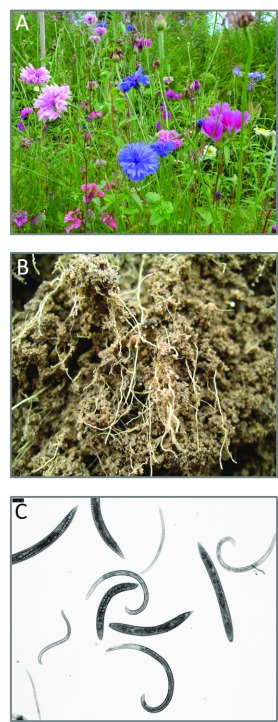
Plant root traits are important drivers of soil food web structure and functioning. ( **a**) Plant communities often consist of multiple coexisting and interacting species varying in values of functional traits such as the quantity and chemical composition of litter they return to the soil. ( **b**) Root nutrient acquisition traits (for example, associations with nitrogen-fixing bacteria or mycorrhizal fungi), architectural habitat traits (for example, root depth, diameter, and branching pattern), and chemical traits (for example, carbon-to-nitrogen ratio and defense compounds) influence the composition and diversity of root-associated organisms and their consumers and predators. ( **c**) Taxonomically and functionally diverse soil nematode communities are often used as indicators of soil food web structure and functioning. Soil nematodes can be allocated to feeding groups, composed of plant feeders (those who live and feed inside plant root tissue and those who feed externally from outside the root), bacterial and fungal feeders, omnivores, and predators. Photos: ( **a**) anthropogenically constructed plant community at Tomtebo Koloniområde, Umeå, Sweden; ( **b**) roots from mixed plant communities after harvest of a greenhouse experiment studying plant-soil feedbacks in old-field succession in the Netherlands ^31^; ( **c**) free-living soil nematodes extracted from soils from a possum exclosure experiment in the Kokatahi River valley in the western South Island of New Zealand ^32^. Photo credits: Paul Kardol.

In recent years, it has been shown that individual plant species differ in their effects on the soil communities they support ^19, 22– 24^. This implies that anthropogenic shifts in plant community composition could have major impacts on soil web structure, as has been shown for urban green spaces, for example ^25^. Here, we follow a simple framework describing three mechanistic pathways of how shifts in plant community composition drive soil food webs ( Figure 2). First, plant species strongly differ in the quantity and quality (that is, the chemical composition) of leaf and root litter they return to the soil ( Figure 3a). The chemical composition of plant litter determines its quality as a resource for detritus feeders and decomposer microbes ^26– 30^. As such, litter quality has often been indicated as a main driver of the relative importance of fungi and bacteria in decomposition processes. Fungi are better able to digest complex, recalcitrant organic compounds (for example, condensed tannins and lignin), and bacteria are more specialized to break down simple, labile organic compounds (for example, sugars) ^33^. However, this traditional view has recently been challenged, and evidence has emerged that fungi may use organic compounds that are more labile than previously expected ^34^. Second, there has been increasing interest in exploring how live plant roots affect soil food webs ( Figure 3b). Root chemistry determines its attractiveness to soil pathogens and herbivores ^35^, and root exudates are important in structuring microbial rhizosphere communities ^36– 38^. Third, plant species could affect soil organisms, and hence soil food web structure, through their effects on soil microclimate and abiotic properties ^39^. For example, plant species differ in their effects on soil moisture, either directly through differences in water uptake or indirectly through effects of shading. Plants can also influence soil organisms through their effects on soil chemistry (for example, through nutrient depletion, nutrient mobilization, or the addition of allelopathic chemicals) ^40– 42^. Although each of these three pathways has been studied for individual plant species, these plant-mediated mechanisms are less well understood for plant communities ^22, 43, 44^. We argue that together these three pathways largely explain the responses of soil food web structure and functioning to changes in plant community productivity, diversity, and composition. Finally, we propose that using a trait-based approach to help understand the mechanisms behind these drivers could provide further guidance.

## Trait-based approaches in community ecology

The increased use of functional trait-based approaches in plant community ecology ^45, 46^ provides new avenues for understanding how shifts in plant communities can influence soil food webs. In plant community ecology, aboveground plant functional traits such as specific leaf area, leaf nutrient content, and leaf dry matter content have been widely used in place of taxonomic diversity measures to explain ecosystem processes and function ^47– 50^. Recently, recognition of the importance of root traits has gained increasing attention ^51– 53^, and greater focus has been put on linking root traits such as root dry matter content, nutrient content, and root architecture to soil processes. For example, changes to root traits associated with exudation could shift C allocation in the rhizosphere and have implications for the soil organisms involved in decomposition and C cycling ^54^. Furthermore, biotic root traits that aid in nutrient acquisition, such as arbuscular versus ectomycorrhizal colonization, impact on the nutritional quality and total quantity of shoot and root litter that enters the soil food web ^55^. To further elucidate the functional linkages between plant communities and the soil food web, recent work has developed and applied a trait-based approach to soil microbes ^56, 57^ and soil fauna ^58– 60^. It has been proposed that investigating the relationship between ‘effect traits’ (that is, traits that determine the effect of an organism on other organisms or its abiotic environment; in this case, plant root traits that influence soil biota) and ‘response traits’ (that is, traits that determine how an organism responds to other organisms or its abiotic environment; in this case, soil food web traits that respond to plants) across plant and soil communities could enable better predictions of ecosystem function ^61^.

**Figure 4.**
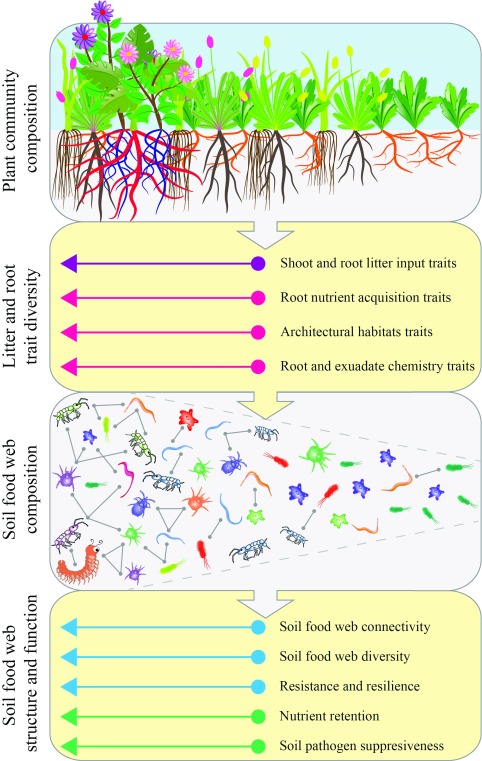
Hypothetical links between shifts in plant community trait composition and soil food web structure and function. Changes in plant community composition result in shifts in community-level values and variation of litter and root traits, which in turn affect the composition, diversity, and connectivity (that is, the number of observed pair-wise interactions expressed as a fraction of the total number of interactions possible) of the soil food web. These changes in the soil food web can have important implications for soil ecosystem functions such as nutrient retention and pathogen suppression. The example shown here illustrates a shift from a simple plant community (for example, an early-successional monoculture) toward a diverse, complex plant community (for example, late-successional, species-rich grasslands or shrubland). Here, the plant community shifts from low to high trait packing and diversity; that is, in simple plant communities, some trait space will be unoccupied, whereas in complex plant communities, the higher diversity of species covers a much wider variety of trait space, leaving little trait space unoccupied. For example, with an increase in plant diversity during long-term ecosystem development in Western Australian shrubland, the diversity of plant nutrient acquisition strategies increases, and almost all nutrient acquisition strategies currently known are represented in the most diverse plant communities ^41, 72, 73^. Diverse, tightly packed plant trait space promotes a greater abundance and diversity of soil biota, connectivity among soil biota, retention of soil nutrients, and resistance and resilience against disturbances. (See Introduction for further explanation.) For example, greater diversity of soil biota may increase soil food web resilience against drought ^13^. We note that plant communities do not necessarily shift from ‘simple’ to ‘complex’ or vice versa; other compositional changes (such as changing composition during succession or plant invasions) with consequent shifts in trait composition are possible. Therefore, our framework can also be applied to shifts in plant trait composition other than those associated with trait packing and diversity per se.

In trait-based ecology, there is often a strong focus on community-weighted mean traits (that is, community-level trait values weighted by species abundances) ^62^. However, in affecting soil food web complexity and diversity, trait variability (that is, the range of variation in root and litter traits) is probably at least as important as community-weighted mean trait values. Therefore, to better understand how plant community trait composition affects the soil food web, we use the concept of trait packing and diversity. High trait packing in a plant community means a high diversity or variation in litter and root traits, leading to more complex, diverse, and stable soil food web structure and function ( Figure 4). If a strong relationship exists between root and litter trait packing in the plant community and characteristics of the soil food web, this might translate to predictable responses in soil ecosystem function. ** For example, it has been shown that microbial community enzyme traits (that is, traits that help break down organic molecules) strongly control litter decomposition rates, which are determined in part by the substrate quality (for example, N content) available to the microbes ^63^. Therefore, inputs of chemically and structurally highly diverse litter, due to high trait packing within the plant community, could foster the development of a trait-packed microbial community and a more diverse soil food web that could help maintain the delivery of multiple ecosystem functions related to nutrient and C cycling and plant productivity. Furthermore, changes to plant community trait composition that affect indirect interactions initiated by belowground predators (that is, behavioral traits) could change the productivity and defense strategy traits of soil organisms on lower trophic levels in ways that affect soil food web connectivity ^64^, which is important because more tightly connected soil food webs are known to promote nutrient retention ^9^.

Below, we explore this framework of trait packing and diversity and, more generally, shifts in litter and root trait values. We focus on areas of research that illustrate how anthropogenic disturbances can affect plant community trait values, leading to shifts in soil food webs. Specifically, we focus on (1) land-use change and secondary succession, (2) climate change and species loss, and (3) plant invasions and range shifts because they are all topical areas of research that are heavily driven by anthropogenic disturbance. We show that under these different scenarios, changes to plant community traits can generate major shifts in the soil food web, leading to positive or negative effects on how soil ecosystems function.

## Land-use change and secondary succession

Plant trait shifts associated with agricultural practices strongly drive soil ecosystem functions. Crop residues (that is, litter traits) and crops with contrasting root traits can have major impacts on soil food web functioning ^50, 65– 67^. For example, root exudate chemical traits have been shown to slow down soil microbial processes, and cereal crops cause slower phosphorus mineralization compared with legumes and this is potentially because of differences in exudate chemical composition ^68^. Crop species and varieties may also strongly vary in root nutrient acquisition strategy, root chemical composition, and root architectural traits (for example, branching patterns) ^69^. Although studies so far have focused mostly on coarse traits, such as C:N ratio and specific root length, such trait differences can impact upon microbial communities ^68^ and higher trophic levels of the soil food web ^70^. Crop species also vary in their attractiveness to soil pests and pathogens, such as host-specific nematodes, because of their distinct root chemistry traits ^71^. Given these inter-specific differences in crop traits, moving from monoculture cropping to mixed cropping would add traits to the system, thereby increasing trait packing and leading to positive effects on soil food web diversity and functioning ( Figure 4). Mixed cropping or using cover crops ensures higher inputs of organic matter into the soil ^74^ and increases the diversity of food sources available for different members of the soil food web ^75, 76^. This, in turn, stimulates the activity and diversity of soil organisms ^10, 75, 77^ and might improve N retention ^78^. For example, using nematode communities as indicators for soil food web structure ( Figure 3b), Leslie *et al*. ^79^ showed that cover crops increased soil food web complexity. Similarly, Chauvin *et al*. ^65^ showed how incorporating litter from three cover crops with contrasting biochemical characteristics into a banana field affected microbe-nematode food webs, and the two litters that were most rich in labile compounds (polysaccharides) increased fungi and bacteria and those nematode groups that fed upon them. Interestingly, these two litters were also most effective in suppression of plant-parasitic nematodes. Taken together, agricultural practices that promote plant trait packing and diversity will likely generate higher connectivity in the soil food web, which will lead to increased resistance and resilience to anthropogenic disturbances in cropping systems ^80^.

A relatively large body of research has focused on how shifts in plant community composition after conversion of agricultural land to (semi)natural systems affect soil food webs and their functioning ^2, 8, 9, 81^. Depending on the management and grazing intensity after abandonment, plant communities typically develop toward species-rich grassland or forest ^81– 83^. Successional changes in plant community composition result in important shifts in litter and root traits, and increases in plant diversity result in more trait packing ( Figure 4). For example, Prieto *et al*. ^84^ showed clear shifts in morphological and chemical root traits across a land-use intensity gradient from disturbed annual crop communities to undisturbed forests. Specifically, fine roots increased in C and lignin concentration and decreased in specific root length with decreasing land-use intensity ^84^. In other words, root trait spectra shifted from a resource acquisition to a conservation strategy. Shifts in plant trait spectra associated with land-use change strongly impact on soil food webs ^2, 85, 86^. Morriën ^6^ nicely illustrated how soil food webs change during secondary succession after cessation of agricultural land use. Notably, increased input of litter with high concentrations of recalcitrant organic compounds stimulated detritus feeders and microbes specialized in breaking down complex organic compounds, and concomitant declines in soil nutrient availability helped promote soil food web connectivity (that is, stronger trophic interactions and increased tightening of the networks of soil biota) ^6, 9^. Furthermore, increased dominance of slow-growing, later-successional plant species, which more strongly depend on associations with mycorrhizal fungi than early species, could shift the fungal community from fast-growing and pathogenic species to slower-growing, beneficial species ^87^. This could affect the rate of C flow through the soil food web ^8^.

## Climate change and species loss

Climatic changes driven in part by anthropogenic activities can strongly influence plant community composition. An increasing number of studies have shown how plant traits are related to climatic adaptation ^88, 89^ and how climate-induced changes in plant community composition can cause major shifts in root and litter trait spectra (for example, for traits that drive water-use efficiency and temperature sensitivity) ^90– 92^. Warming affects plant physiology and phenology and ultimately can result in altered plant dominance and shifts in range distributions of plant species (see ‘Plant invasions and range shifts’ section below). However, changes in precipitation regime, such as longer and more intense droughts, could be expected to most dramatically affect plant community trait spectra, at least in short to moderate timescales ^93^. For example, along an aridity gradient, root tissue density and specific root length may shift to more conservative values with increasing aridity, and the diversity of acquisition trait values may increase, facilitating a wider array of resource acquisition strategies under conditions of water stress ^94^. In old-field communities, experimental drought shifted plant cover dominance from a woody, N-fixing sub-shrub to a C3 bunchgrass and had far-reaching consequences for soil food web structure. Moreover, microbial enzyme activities and nematode feeding group composition indicated higher soil food web complexity but slower rates of nutrient cycling in soils beneath the sub-shrub compared with the grass, most likely because of high concentrations of polyphenolics and lignin in organic residues from the sub-shrub ^43^. In general, drought- and other climate-induced changes in plant trait spectra could greatly modify or counteract direct climate impacts on the soil food web ^43, 95, 96^.

Climate change not only may alter plant species composition but also can result in species loss ^97, 98^. In turn, loss of species from the plant community will lower litter and root trait diversity and packing ( Figure 4). Although we are not aware of any studies explicitly testing how decreased trait packing under climate change would affect soil food webs, we can use plant species removal and biodiversity manipulation experiments to infer the consequences. Removal of plant functional groups in grasslands has shown that decreased functional group richness generally lowers the abundance of primary decomposers (microbes) and their consumers (nematodes), and these effects are strongest when the most dominant plant functional groups are removed ^99^. Loss of plant functional groups also decreased the ratio between bacterial- and fungal-feeding nematodes, which could be partly linked to shifts in nematode food resources. These shifts in soil food web composition in response to plant functional group loss could be associated with lower nutrient and C retention in the soil ^99^. Effects of plant functional group removal on soil food web components in the boreal forest depended on plant group dominance but could generally be explained by reductions in the quantity and quality of plant litter input to the soil ^100, 101^. Essentially, the loss of highly labile (that is, nutrient-rich) litter inputs caused by deciduous shrub removal may have detrimentally impacted on the microbial and nematode communities because these two groups are highly dependent on such inputs as both direct and indirect food sources ^100, 101^. For randomly assembled plant communities, the effects of lower plant species and functional group richness on soil biota are mostly negative but weaker for soil biota occupying higher levels in the soil food web ^102^. For nematodes, these effects of plant species and functional group diversity have been linked to changes in litter quality (that is, plant shoot C:N ratio) ^103^, but potential effects mediated through shifts in root nutrient acquisition, architectural, and chemical traits remain to be tested.

## Plant invasions and range shifts

Exotic invasive plant species introduced by humans are altering plant community composition across the globe ^104^, and debate concerning the consequences of plant invasions for ecosystem functioning continues ^105^. Invasive plants generally have higher values for traits associated with growth rate, tissue nutrient content, and competitive ability (that is, production of allelopathic chemicals in litter or root exudates) compared with natives ^106, 107^. Therefore, invasive plants can introduce novel traits into the existent plant community that could affect the soil food web. For example, allelopathic chemicals produced by the invasive tree *Ailanthus altissima* can hinder soil microbial activity and thereby nutrient mineralization, while high litter production can increase earthworm abundance, potentially offsetting this negative effect ^108^. Furthermore, invasion by the forb *Solidago gigantea* increased fungal biomass and had disproportionate cascade effects on certain fungal-feeding nematode taxa that were probably due to disparate feeding abilities among the nematodes ^109^. In contrast, invasion by a grass resulted in less allocation of C to higher trophic levels of soil nematodes compared with a native grass species ^110^. Taken collectively, traits associated with contrasting functional groups of invasive plants (that is, trees, forbs, and grasses) could lead to reduced trait packing ( Figure 4), thereby minimizing the complexity of the soil food web by leading to the dominance of certain groups of soil organisms. This could reduce the ability of the soil food web to cycle nutrients ^8^ and stifle its resistance to disturbance. These findings highlight that invasive plants can bring new traits into a system, which might impact disproportionately on different groups of soil organisms, leading to alterations of functions provided by the soil food web.

Furthermore, expansion of plant species into previously un-colonized ranges (as expected under global warming; see ‘Climate change and species loss’ section above) has the potential to introduce new species with new traits into the community and have repercussions for the soil food web. The widening of niche envelopes (that is, the environmental conditions necessary for occupation by a certain species) that leads to range expansion ^111^ could result in unique interactions between plant communities and the soil food web. For example, Wilschut *et al*. ^112^ showed that range-expanding forbs could exert bottom-up control on root-feeding nematodes (likely through novel allelopathic chemicals exuded from their roots) but that native congeners tended to use top-down control through changes to the microbial community. This finding corroborates the novel weapons hypothesis ^113^ and showcases the role that range-expanding plant traits can play in changing the soil food web. Range-expanding plants might also escape their enemies in the soil food web (that is, the enemy release hypothesis) ^114^, and this, combined with favorable climatic conditions, could lead to successful establishment ^115^. Furthermore, range-expanding plants might fail to find suitable decomposer organisms for their litter (that is, lack of home-field advantage effects) ^116^ because of mismatches in litter chemistry traits and soil organisms specialized in breaking down this litter. Finally, range-expanding plants may not establish mycorrhizal associations (that is, incompatible root nutrient acquisition traits; Figure 4) ^117^, potentially leading to failed colonization ^118^. However, there is a lack of empirical evidence for these effects and further studies are needed to understand how range expansion impacts on trait packing in the plant community and thereby the soil food web ( Figure 4).

## Conclusions

Anthropogenic shifts in plant community composition and diversity are likely to have major implications for the composition and function of soil food webs as well as the services they provide. Much recent progress has been made, and our trait-based conceptual model provides guidance for future studies to elucidate the underlying mechanisms of how shifts in plant community traits could lead to cascade effects belowground. The following areas in particular warrant future attention: (1) We know relatively well how functional differences among individual plant species affect soil food webs, but much less is known about the effects of complex plant communities where multiple species coexist and interact. Here, it would be of interest to separate the effects of community-weighted mean values from the diversity of traits represented in the community. (2) The majority of studies inferring changes in soil food web functioning focus exclusively on microbes or use soil nematode communities as indicators of soil food web structure. These approaches have yielded important insights, but to fully understand the role of soil food webs in how shifts in plant community composition affect soil ecosystem functioning, we need to look at whole soil food webs, including organisms at higher trophic levels. (3) Knowledge about the quality and quantity of substrate required by soil microbes and fauna is increasing, and ideas about interactions between different trophic levels are being revised. However, further studies are needed to understand the complex transferring of energy between the different organisms in the soil food web. Therefore, it is integral to investigate how energy transfer within the soil food web is driving key ecosystem processes and to focus particularly on the traits involved. (4) Plant trait-based research has seen a steep increase in activity in recent years, including new research explicitly focusing on root traits. However, the traits most commonly used in these studies are not always the most meaningful in terms of their importance for the functioning of soil communities. Instead of focusing on coarse traits, such as C:N ratios of shoots and roots, it would be more ecologically informative to look at the molecular construction of plant-derived C and N compounds, such as phenolics and their derivatives, which are known drivers of soil microbial activity and resource use efficiency ^95^, which link more strongly to ecosystem processes and function. (5) Soil food webs often respond slowly and show remarkable resistance to environmental changes. Hence, the effects of shifts in plant community composition may become apparent only at larger timescales. This requires long-term studies and awareness of long-lasting soil legacies. (6) Many studies exploring the relationships between plant communities and soil food webs use observational approaches ^9, 81, 119– 122^. Although observations allow coverage of large spatial and temporal scales (that is, chronosequences), these studies do not disentangle the mechanisms. We advocate for additional empirical studies explicitly manipulating litter and root trait spectra and diversity. Only through continued research will we be able to better understand how anthropogenically driven shifts in plant community composition will affect complex soil food web interactions and the ecosystem services that they provide.
